# Genotoxicity of pyrrolizidine alkaloids in metabolically inactive human cervical cancer HeLa cells co-cultured with human hepatoma HepG2 cells

**DOI:** 10.1007/s00204-022-03394-z

**Published:** 2022-10-23

**Authors:** Naji Said Aboud Hadi, Ezgi Eyluel Bankoglu, Helga Stopper

**Affiliations:** 1 Institute of Pharmacology and Toxicology, University of Wuerzburg, Versbacher Straße 9, 97078 Würzburg, Germany; 2 School of Health and Human Sciences, Pwani University, Kilifi, Kenya

**Keywords:** Co-culture, Micronuclei, Mitotic disturbance, Cytochrome P450s, Membrane transporters, Pyrrolizidine alkaloids

## Abstract

Pyrrolizidine alkaloids (PAs) are secondary plant metabolites, which can be found as contaminant in various foods and herbal products. Several PAs can cause hepatotoxicity and liver cancer via damaging hepatic sinusoidal endothelial cells (HSECs) after hepatic metabolization. HSECs themselves do not express the required metabolic enzymes for activation of PAs. Here we applied a co-culture model to mimic the in vivo hepatic environment and to study PA-induced effects on not metabolically active neighbour cells. In this co-culture model, bioactivation of PA was enabled by metabolically capable human hepatoma cells HepG2, which excrete the toxic and mutagenic pyrrole metabolites. The human cervical epithelial HeLa cells tagged with H2B-GFP were utilized as non-metabolically active neighbours because they can be identified easily based on their green fluorescence in the co-culture. The PAs europine, riddelliine and lasiocarpine induced micronuclei in HepG2 cells, and in HeLa H2B-GFP cells co-cultured with HepG2 cells, but not in HeLa H2B-GFP cells cultured alone. Metabolic inhibition of cytochrome P450 enzymes with ketoconazole abrogated micronucleus formation. The efflux transporter inhibitors verapamil and benzbromarone reduced micronucleus formation in the co-culture model. Furthermore, mitotic disturbances as an additional genotoxic mechanism of action were observed in HepG2 cells and in HeLa H2B-GFP cells co-cultured with HepG2 cells, but not in HeLa H2B-GFP cells cultured alone. Overall, we were able to show that PAs were activated by HepG2 cells and the metabolites induced genomic damage in co-cultured HeLa cells.

## Introduction

Pyrrolizidine alkaloids (PAs) are phytotoxins occurring naturally in about 3% of all flowering plants worldwide (European Medicines Agency 2021; Edgar 2002). PAs have been found as cross-contaminants in various human consumption products such as spices, herbal teas and herbal medicines (Fu et al. 2007; Kakar et al. 2010; Risk-Assessment 2013; Zhu et al. 2018). Hundreds of PAs have been described up to now and it is assumed that about half of them are hepatotoxic (Fu et al. 2004; Hessel-Pras et al. 2020; Stegelmeier et al. 1999; Xiaobo He 2018).

**Fig. 1:**
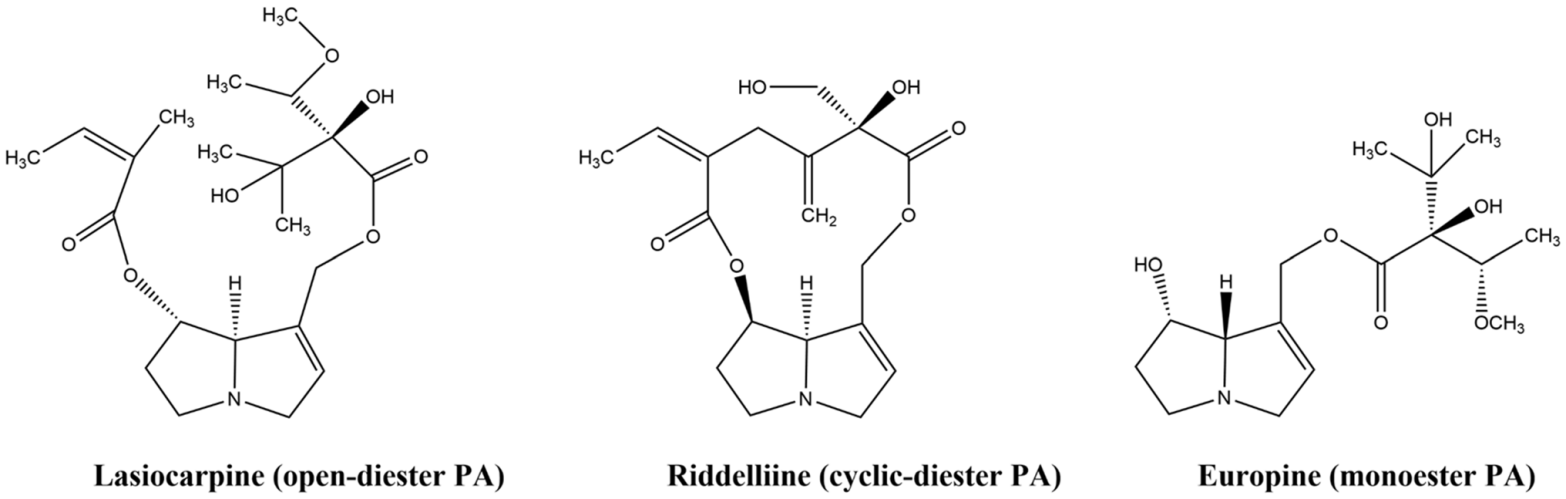
Examples of pyrrolizidine alkaloids (PAs) based on their ester type. The chemical structures were created using ChemDraw 19.0 (version 2019)

PAs can be categorized based on their esterification at the necic acid moiety. Examples are the monoester europine, the cyclic diester riddelliine or the open diester lasiocarpine (Fig. 1). They require metabolic activation in liver hepatocytes by cytochrome P450 enzymes to form reactive pyrrolic metabolites, which can bind to cellular proteins to form pyrrole–protein adducts and cause cytotoxicity (He et al. 2021b; Ma et al. 2019, 2021). These reactive pyrrolic metabolites can also bind to cellular DNA to form pyrrole–DNA adducts and induce genotoxicity (He et al. 2021a; Xia et al. 2013; Zhu et al. 2017) or conjugate with the reduced form of glutathione to form pyrrole–GSH conjugates which are then excreted via the urinary or biliary way (Lin et al. 1998, 2000) or may contribute further to genotoxicity.

Human exposure to PAs has been considered as one of the primary causes of hepatic sinusoidal obstruction syndrome (HSOS), earlier known as hepatic veno-occlusive disease (Xu et al. 2019). At an early stage, HSOS is characterized by primary hepatic sinusoidal endothelial cell (HSEC) damage. HSECs are also the main target cells of PA-induced carcinogenicity (DeLeve et al. 2002, 2003; Lu et al. 2019). However, the mechanism has not yet been fully elucidated because liver HSECs lack metabolic enzymes that are required to activate PAs.

Due to human health and safety concerns, several studies have been performed recently to investigate the mutagenicity of PAs (Chain 2011; Chen et al. 2019, 2010; Risk-Assessment 2013). For example, PAs of different ester types induced genotoxicity in HepG2 cells and differed in their potency with open diester PAs inducing significant micronucleus formation at the lowest concentration, followed by cyclic diesters and then monoester PAs (Allemang et al. 2018; Hadi et al. 2021; Louisse et al. 2019). In addition, DNA cross-linking activity of equimolar concentrations of PAs was significant for diester Pas, while monoester PAs only yielded a non-significant effect (Hadi et al. 2021).

The present study uses a co-culture cell model to investigate the mode of action of PAs further. For this, non-metabolically active HeLa H2B-GFP cells and metabolically active HepG2 cells were cultured together. HeLa H2B-GFP cells are human cervical epithelial cancer cells that express green fluorescent protein-fused histone H2B (H2B-GFP) and they have been widely used to visualize the dynamics of chromosomal abnormalities in living cells during various processes (Huang et al. 2011; Kanda et al. 1998; Reimann et al. 2020). HepG2 are human-derived hepatocarcinoma cells which are known to express metabolic enzymes (Donato et al. 2015; Hadi et al. 2021). This co-culture system mimics the presumed in vivo situation where liver hepatocytes activate PAs, but the target cells for toxicity and mutagenicity are the metabolically inactive HSECs cells.

## Materials and methods

### Chemicals and reagents

PAs (europine (CAS# 570-19-4; assay 100%), riddelliine (CAS# 23,246-96-0; assay ≥ 98%) and lasiocarpine (CAS# 303-34-4; assay 100%) were obtained from PhytoLab (Vestenbergsgreuth, Bayern, Germany); Cyclophosphamide (CAS# 6055–19-2; assay ≥ 97%) was from Alfa Aesar (Karlsruhe, Germany). Gel Green Nucleic Acid stain was obtained from Biotium (Darmstadt, Germany). Fluorescein diacetate (FDA; CAS# 596-09-8) was from Invitrogen (Germany). Dimethylsulfoximide (DMSO; ≥ 99.8%), bisBenzimide H33258 (CAS# 23,491–45-4; assay ≥ 98%), diazabicyclo-octane (DABCO), ketoconazole (CAS# 65,277-42-1; assay ≥ 98%), quinidine (CAS# 56-54-2; assay ≥ 97%), verapamil (CAS# 152-11-4; assay ≥ 99%), nelfinavir (CAS# 159,989-65-8; assay ≥ 98%), benzbromarone (CAS# 3562-84-3; assay ≥ 95%), vincristine (CAS# 2068-78-2; assay ≥ 97%), cytochalasin B (CAS# 14,930-96-2), ethidium bromide (CAS# 1239-45-8; assay ≥ 95%) and sodium fluorescein (CAS# 518-47-8; assay ≥ 95%) were from Sigma-Aldrich (Steinheim, Germany). Calcein-acetoxymethyl ester (Calcein-AM; CAS# 148,504-34-1; assay ≥ 95%) was from Cayman Chemical Company (Germany). Cell culture media and reagents were all from Sigma-Aldrich (Steinheim, Germany), except foetal bovine serum, which was from Biochrom (Berlin, Germany).

### Cell lines and co-culture model

The human hepatoma cells HepG2 (doubling time 40 h) were cultured at 37 °C with 5% (*v*/*v*) CO_2_ in minimum essential medium (MEM) supplemented with 10% (*v*/*v*) foetal bovine serum, 1% (*v*/*v*) L-glutamine, 1% (*v*/*v*) antibiotics (50 U/mL penicillin and 50 mg/mL streptomycin) and 1% (*v*/*v*) nonessential amino acids. Cells were sub-cultured twice per week.

Human cervical cancer epithelial cells (HeLa; doubling time 20 h) that express green fluorescent protein-fused histone H2B (HeLa H2B-GFP) were obtained from Henning Hintzsche, University Bonn, Germany, and Noriaki Shimizu, Graduate School of Integrated Sciences for Life, Hiroshima University, Japan (Kanda et al. 1998; Reimann et al. 2004; Utani et al. 2010), and were cultured at 37 °C with 5% (*v*/*v*) CO_2_ in high-glucose Dulbecco’s modified Eagle’s medium without phenol red, but supplemented with 10% (*v*/*v*) foetal bovine serum, 1% (*v*/*v*) L-glutamine, 1% (*v*/*v*) sodium pyruvate solution, 1% (*v*/*v*) HEPES sodium salt solution and 1% (*v*/*v*) antibiotics (50 U/mL penicillin and 50 mg/mL streptomycin). Cells were sub-cultured thrice per week.

The co-culture model was set up such that overgrowth of the faster proliferating HeLa H2B-GFP cells over HepG2 cells was prevented within the duration of the experiment. The applied conditions for co-culture were that HepG2 cells and HeLa H2B-GFP cells were both seeded together in a six-well plate at 40,000 HeLa H2B-GFP cells and 150,000 HepG2 cells per well with 3 mL of HepG2 culture medium, and then incubated at 37 °C with 5% (*v*/*v*) CO_2_ overnight, to allow the cells to settle and attach to the plate.

### Cytokinesis-block micronucleus (CBMN) assay

The applied concentrations of the different ester type PAs lasiocarpine (10 µM; open diester PA type), riddelliine (100 µM; cyclic diester PA type) and europine (320 µM; monoester PA type) were based on our previously published study (Hadi et al. 2021) choosing concentrations at which significant induction of micronuclei were achieved. The study of genomic damage was performed in a six-well plate in which one well consisted of only HepG2 cells, one well of only HeLa H2B-GFP cells and further wells containing both cell types in co-culture. To achieve this, 190, 000 of HeLa H2B-GFP cells per 3ml, 150,000 of HepG2 cells per 3ml and 40,000 HeLa H2B-GFP plus 150,000 HepG2 for the co-culture per 3ml were seeded and incubated overnight. As culture medium, the HepG2 cell medium was used for all cells. After 24 h, the culture medium was renewed, followed by treatment of the cells with solvent control (DMSO), lasiocarpine, riddelliine or europine for 28 h. Then, the medium was discarded again, cells washed with PBS, followed by adding fresh medium with 3 µg/ml cytochalasin B for 20 h in HeLa H2B-GFP cells and co-culture, while the culture only containing HepG2 cells was incubated with cytochalasin B for 44 h due to their longer doubling time compared to HeLa H2B-GFP cells. Thereafter, the cells were harvested and microscopic slides (76 × 26 × 1 mm; Marienfeld GmbH & Co.KG) were prepared by cytospin centrifugation (Cytospin3 centrifuge, Thermo Shandon). Then, the slides were fixed in − 20 °C methanol for at least 2 h, after which the slides with co-cultures and HeLa H2B-GFP cells were mounted without application of a dye with diazabicyclo-octane (DABCO) to evaluate the micronucleus formation in HeLa H2B-GFP cells. The slides with only HepG2 cells were stained with 10 µL GelGreen Nucleic Acid solution (1:100 dilution in bi-distil water) for 6–7 min, washed with PBS and then mounted with DABCO. At this point, slides were coded and for each test sample micronuclei were scored in 1000 binucleated HeLa H2B-GFP cells in the co-culture and HeLa H2B-GFP cells, and in 1000 binucleated HepG2 cells in the HepG2 cell culture, at 400-fold magnification with an Eclipse 55i fluorescence microscope using a fluorescein isothiocyanate (FITC) filter (Nikon GmbH, Japan). The numbers of mononucleated cells (MN), binucleated cells (BN), trinucleated cells (TriN), and tetranucleated cells (TetraN) in 1000 cells were also scored. The cytokinesis-block proliferation index (CBPI) was calculated from that as a criterion for determining the cytotoxic effect using the following formula:

$${\text{CBPI}}\, = \,\frac{{\left( {1\, \times \,{\text{MN}}} \right)\, + \,\left( {2\, \times \,{\text{BN}}} \right)\, + \,\left( {3\, \times \,{\text{TriN}}} \right)\, + \,\left( {4\, \times \,{\text{TetraN}}} \right)}}{{{\text{MN }}\, + \,{\text{ BN}}\,{ } + \,{\text{ TriN }}\, + \,{\text{ TetraN}}}}.$$

Data of all micronucleus experiments are shown as average with standard deviation from at least three independent repeat experiments.

### Application of inhibitors of metabolic enzymes and membrane transporters

The cytochrome P450-3A4 isoenzyme inhibitor ketoconazole (1 µM) was applied as a pretreatment for 24 h to the co-cultured cells (Araki et al. 2012; Novotna et al. 2014; Weemhoff et al. 2003; Westerink and Schoonen 2007). Then, the PAs lasiocarpine, riddelliine and europine were applied and the further procedure was followed as described above for the micronucleus assay. Since the analysis was limited to HeLa H2B-GFP cells in these experiments, cytochalasin B (at final concentration 3 µg/ml) exposure was 20 h.

Transmembrane transporters may enhance or decrease PA-mediated effects. In the co-culture model system, the efflux membrane transporters were inhibited based on published effective concentrations of known inhibitors. The idea was that metabolites formed in hepatocytes may not reach HeLa H2B-GFP cells to the same extent. The multidrug resistance protein 1 (MDR1) inhibitor verapamil (50 µM; (Muller 1995; Donmez et al. 2011; Louisa et al. 2016; Nobili 2006)), and the multidrug resistance–associated protein 2 (MRP2) inhibitor benzbromarone (10 µM; (Huisman 2010; Sinclair and Fox 1975)) were applied as a pretreatment for 24 h in the co-culture model. Then, the co-culture was exposed to PAs for 28 h, followed by 3 µg/ml cytochalasin B for 20 h. The procedure was as described for the micronucleus assay except that 160 µM europine was used, which yielded a similar amount of micronuclei as the previously used 320 µM.

The activity of the transporter inhibitors was determined prior to these experiments by measuring intracellular accumulation or retention of reference fluorescent substrates such as calcein-acetoxymethyl ester (calcein-AM) as reference substrate of MDR1 (Essodaigui et al. 1998) and calcein as a reference substrate of MRP2 efflux transporter (Bauer et al. 2003; Evers et al. 2000). The activity of MDR1 and MRP2 efflux transporters was significantly reduced to accumulate or retain the calcein fluorescence intensity (green fluorescence) by applying verapamil (50 µM) or benzbromarone (10 µM), respectively (performed in HepG2 cells; data not shown). Since ketoconazole (1 µM), which was used as an inhibitor of cytochrome P450-3A4 (Arzuk et al. 2020; Elsherbiny et al. 2008; Greenblatt 2014; Greenblatt 2016; Greenblatt and Greenblatt 2014; Kalgutkar et al. 2009; Karthik Venkatakrishnan 2000; Li et al. 2014; Novotna et al. 2014; Ohyama et al. 2000; Pelkonen et al. 2008; Vermeer et al. 2016; Zhang et al. 2017), had been reported to inhibit MDR1 efflux transporter with IC_50_ > 6 µM (Nikulin 2017; Vermeer et al. 2016), the cross-activity effect of ketoconazole (1 µM) with efflux transporter (MDR1) in HepG2 cells was also determined and no accumulation of the fluorescent substrates for efflux transporter was observed (performed in HepG2 cells; data not shown).

### Analysis of mitotic figures

The characterization of mitotic cells in fixed cell preparations was performed as described in Baudoin and Cimini (2018). The analysis regarding the numbers of cells in each of the mitotic stages as well as disturbed arrangements in each of the stages was performed using HepG2 cells cultured alone, HeLa H2B-GFP cells cultured alone, and the HeLa H2B-GFP cells of the co-culture similar to the procedure described for micronucleus experiments, except without the incubation with cytochalasin B. Cells were treated and incubated with lasiocarpine (10 µM), riddelliine (100 µM) and europine (160 µM), concentrations which induced a similar amount of micronuclei for each of the PA, vincristine (10 ng/ml; positive control for mitotic disturbance) and solvent controls for 28 h. Thereafter, cells were harvested and the microscopic slides prepared as described for micronucleus analysis. The mitotic index (MI; % cells in mitosis) was assessed by counting and adding up the number of cells in the mitotic stages prophase, metaphase, anaphase and telophase in 1000 cells.

**Fig. 2:**
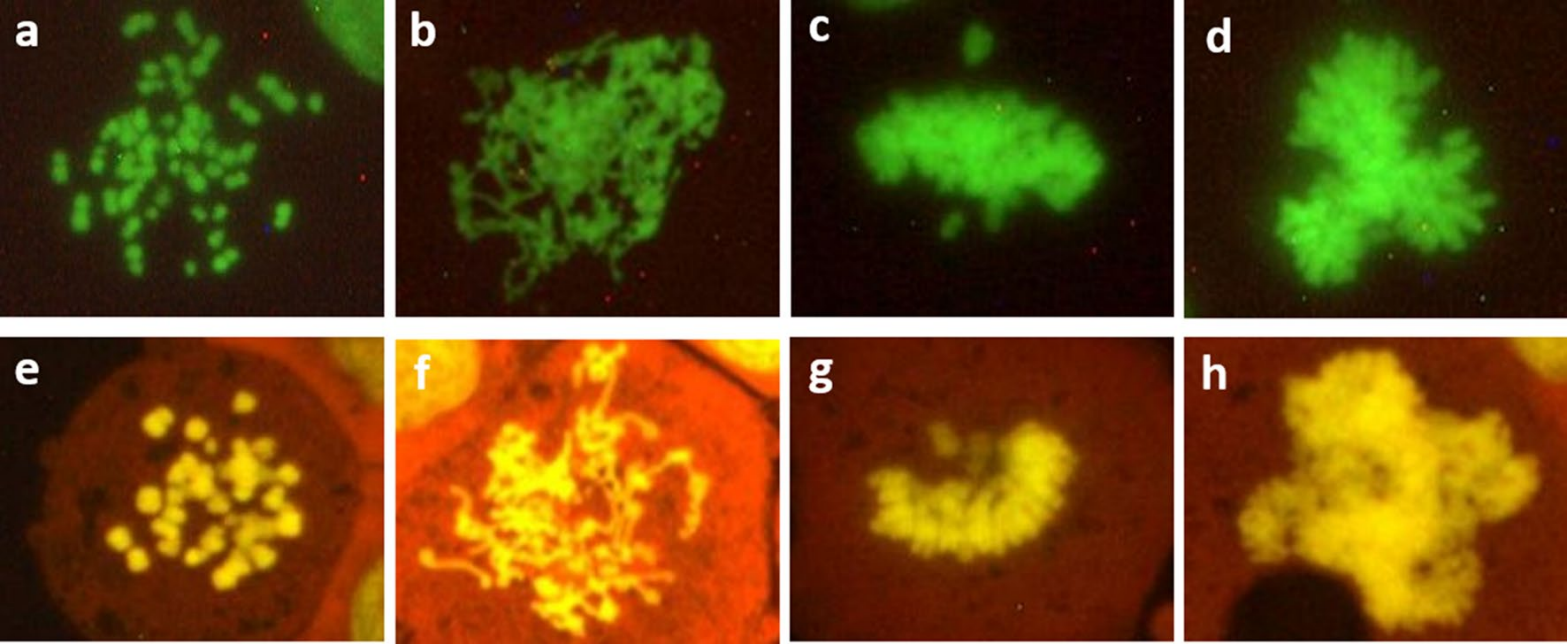
Representative images of metaphase mitotic disturbances in HeLa H2B-GFP cells (**a**, **b**, **c**, **d**) and HepG2 cells (**e**, **f**, **g**, **h**). The cells in co-culture and HeLa H2B-GFP cells were mounted with DABCO without staining, while HepG2 cells were stained with GelGreen and mounted with DABCO. The classified metaphase disturbances are: no spindle formation (**a**, **e**); elongated chromosomes/chromatids (**b**, **f**); non-congression (**c**, **g**); and multipolar metaphase (**d**, **h**)

**Fig. 3:**
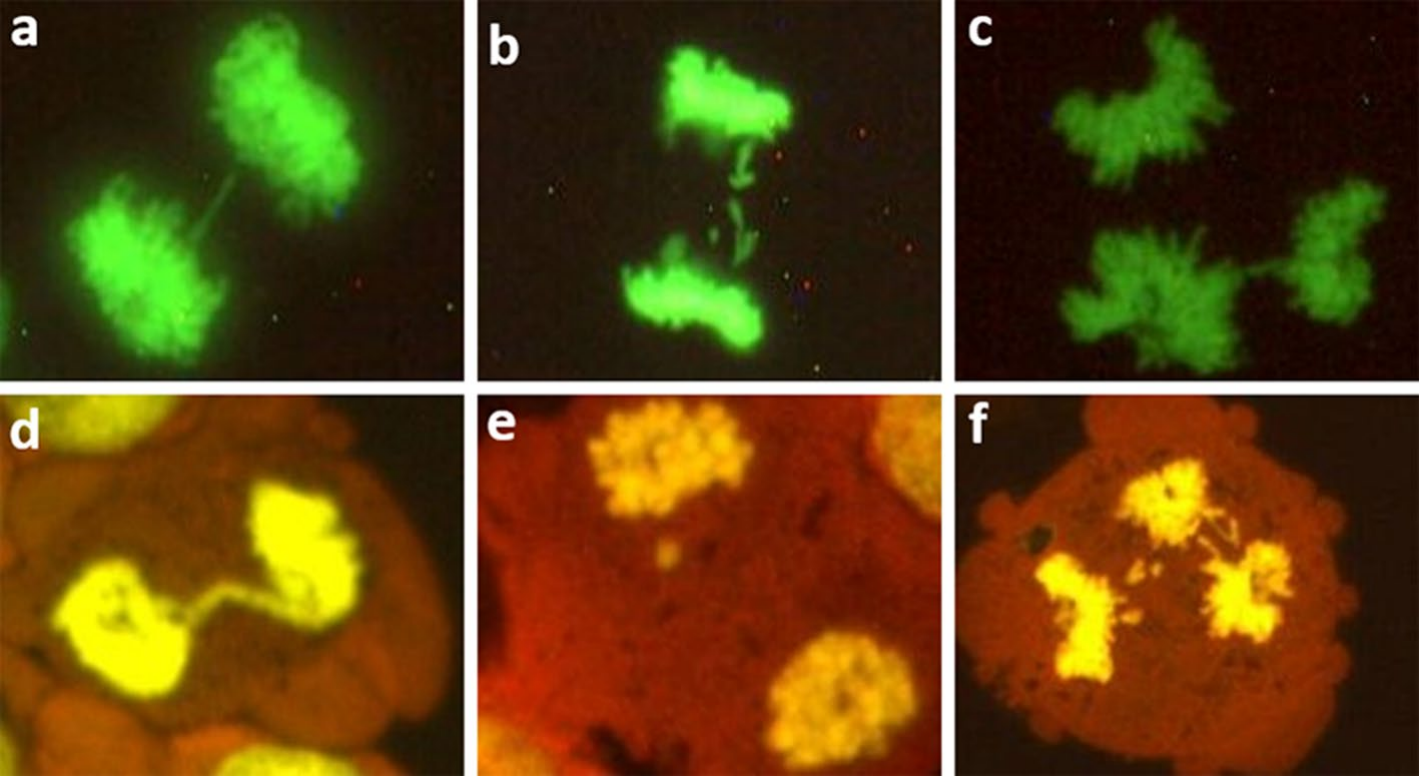
Representative images of anaphase–telophase mitotic disturbances in HeLa H2B-GFP cells (**a**, **b**, **c**) and HepG2 cells (**d**, **e**, **f**). The HeLa H2B-GFP cells in co-culture and HeLa H2B-GFP cells were mounted with DABCO without staining, while the slides with only HepG2 cells were stained with GelGreen and then mounted with DABCO. The classified anaphase–telophase disturbances are: bridges (**a**, **d**); lagging or separate chromosome(s)/chromatid(s) (**b**, **e**); and multipolar anaphase–telophase (**c**, **f**)

Disturbed mitoses were defined as mitotic figures deviating from the typical appearance of mitoses and were assessed in 300 mitotic cells per slide. We classified the mitotic disturbances into the following categories (Figs. 2, 3):

Metaphase: no metaphase plate arrangement, non-congression of individual chromosomes to the metaphase plate, multipolar (tripolar and more than tripolar) arrangement of chromosomes and elongated chromosomes/chromatids.

Anaphase: bridges, lagging chromosome(s), combination of bridges and lagging chromosome, and multipolar (tripolar and more than tripolar) arrangement of chromosomes.

Telophase: same analysis as for anaphase cells.

If a cell harboured more than one type of disturbance, it was added to each category (e.g. multipolar, lagging chromosome(s) and bridges).

Typical sample images of mitotic disturbances are shown in Figs. 2 and 3.

### Statistical analysis

All data are expressed as mean ± standard deviation (SD). One-way ANOVA test and independent sample *T *test (Student’s *T* test) were used for the comparison among various groups and between two groups, respectively. All statistics were performed using either GraphPad Prism version 9.4.0 (GraphPad Software, Inc., USA) or EXCEL version 16. Graphs were created using GraphPad Prism version 9.4.0 (GraphPad Software, Inc., USA). Statistical significance was set at *p* value < 0.05.

## Results

### Induction of micronuclei by selected PAs

**Fig. 4:**
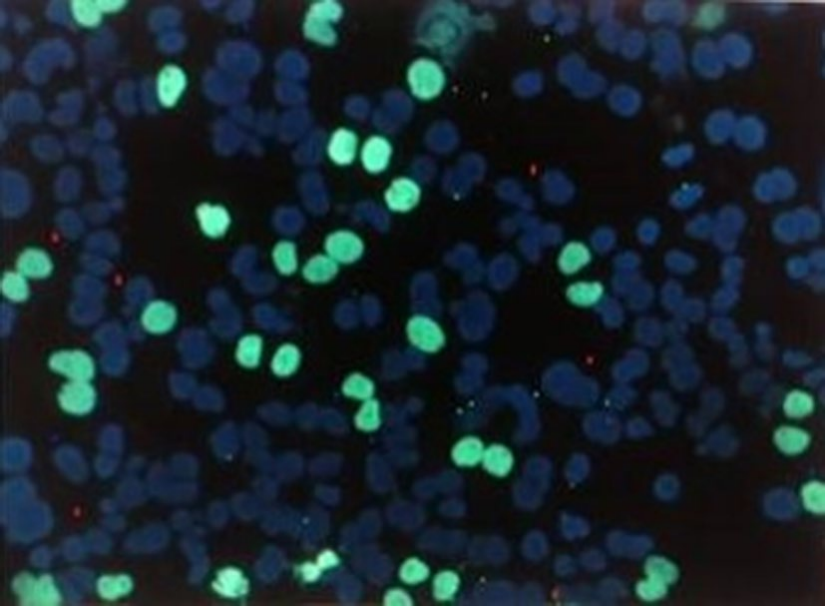
Representative image of the co-culture cell ratio at the time of cell harvest achieved by seeding 40,000 of HeLa H2B-GFP cells (green) and 150,000 of HepG2 cells (blue). Images were taken at × 200-fold magnifications and are shown as overlay of UV excitation and FITC filter. Cells were stained with bisBenzimide (Hoechst 33,342) dye (blue) and viewed under Eclipse 55i florescence microscope

For the co-culture, seeding of 40,000 of HeLa H2B-GFP cells and 150,000 of HepG2 cells yielded a suitable co-culture cell ratio between the two cell lines corresponding to 32.24% HeLa H2B-GFP cells and 67.76% HepG2 cells at the time of harvest (Fig. 4).

**Fig. 5:**
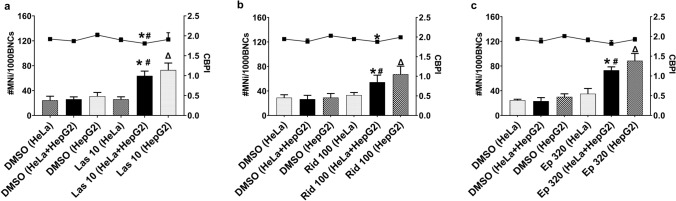
Micronucleus induction (columns) and proliferation index (CBPI; line) in HeLa H2B-GFP cells, co-culture and HepG2 cells after treatment with the indicated pyrrolizidine alkaloids. **a** Lasiocarpine, **b** riddelliine and **c** europine. *p < 0.05 compared to solvent control in HeLa H2B-GFP cells (DMSO; HeLa H2B-GFP). #*p* < 0.05 compared to solvent control in co-culture (DMSO; combination of HepG2 + HeLa H2B-GFP). Δ*p* < 0.05 compared to solvent control in HepG2 cells (DMSO; HepG2). *Las 10*  lasiocarpine 10 µM (open diester PA); *Rid 100* riddelliine 100 µM (cyclic diester PA), *Ep 320* europine 320 µM (monoester PA), *DMSO* dimethyl sulphoxide (solvent control), *MNi *micronucleus, *BNCs* binucleated cells, *CBPI* cytokinesis-block proliferation index; *n* = 3

The quantification of PA-induced micronucleus formation is shown in Fig. 5. There was significant induction of micronucleus formation in HeLa H2B-GFP cells in the co-culture treated with PAs compared to the solvent control. In the culture of only HeLa H2B-GFP, there was no micronucleus formation associated with treatment with PAs, while micronucleus induction was achieved with PAs in cultures of only HepG2 cells.

### Inhibitors of metabolism and membrane transporters in the co-culture

**Fig. 6:**
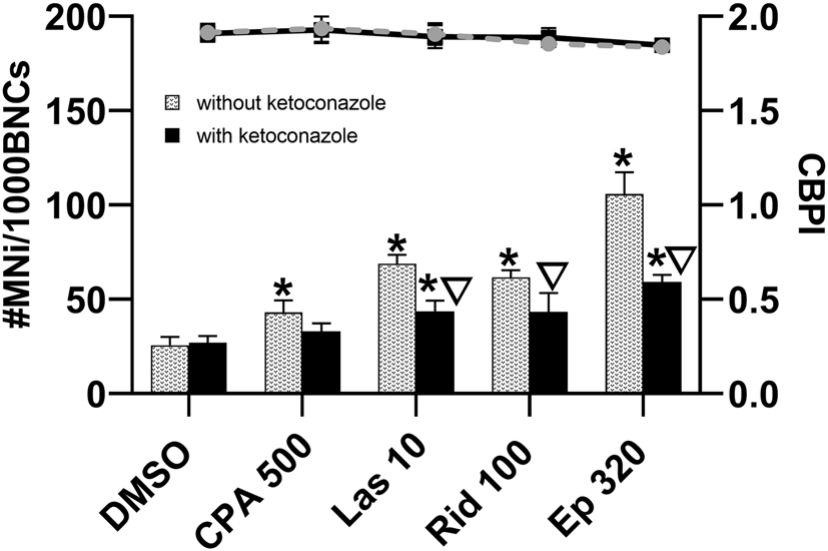
Micronucleus frequency (columns) and proliferation index (CBPI; line) in the co-culture model consisting of HeLa H2B-GFP and HepG2 cells. Cells were pretreated with ketoconazole for 24 h, then treated with PAs for 28 h and compared with the standard protocol without ketoconazole pretreatment. *p < 0.05 compared with solvent control (DMSO), ∇*p* < 0.05 compared with the respective dose without inhibitor pretreatment. *MNi* micronucleus, *BNCs* binucleated cells, *CBPI* cytokinesis-block proliferation index, *CPA* *500* cyclophosphamide 500 µM (positive control), *Las 10* lasiocarpine 10 µM, *Rid 100* riddelliine 100 µM, *Ep 320* europine 320 µM; *n* = 3

Further mechanistic investigation was done in the co-culture model using ketoconazole for cytochrome P450-3A4 isoenzyme inhibition. As shown in Fig. 6, ketoconazole significantly reduced lasiocarpine-, riddelliine- and europine-induced micronucleus formation.

**Fig. 7:**
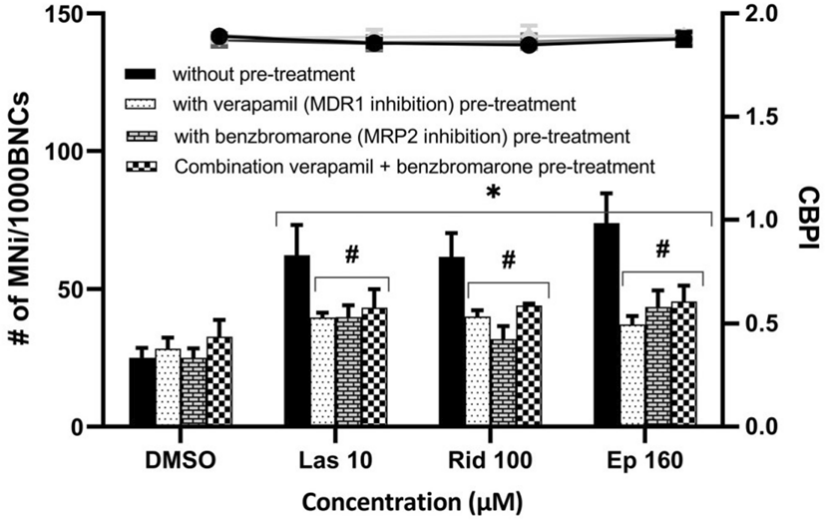
Micronucleus frequency (columns) and proliferation index (CBPI; line) in the co-culture model consisting of HeLa H2B-GFP and HepG2 cells. MDR1 efflux transporter inhibitor verapamil, MRP2 efflux transporter inhibitor benzbromarone and the combination of verapamil and benzbromarone were applied for 24 h. Then, cells were treated with PAs of different ester types for 28 h and compared with their standard protocol without inhibitor pretreatment. *p < 0.05 compared with solvent control (DMSO), ∆*p* < 0.05 compared with the respective dose without inhibitor pretreatment. *MNi* micronucleus, *BNCs* binucleated cells, *CBPI* cytokinesis-block proliferation index, *Las 10* lasiocarpine 10 µM, *Rid 100* riddelliine 100 µM, *Ep 160* europine 160 µM; *n* = 3

Impairing the efflux of metabolites from HepG2 cells should reduce their amount available for uptake into HeLa H2B-GFP cells. Therefore, efflux transporter chemical inhibitors were applied in the co-culture model and the micronucleus formation was determined (Fig. 7). Both, the inhibitor of MDR1 efflux transporter, verapamil, and the inhibitor of MRP2 efflux transporter, benzbromarone, as well as their combination, significantly reduced lasiocarpine-, riddelliine- and europine-induced micronucleus formation in HeLa H2B-GFP cells within the co-culture with HepG2 cells.

### Analysis of mitotic figures

**Fig. 8:**
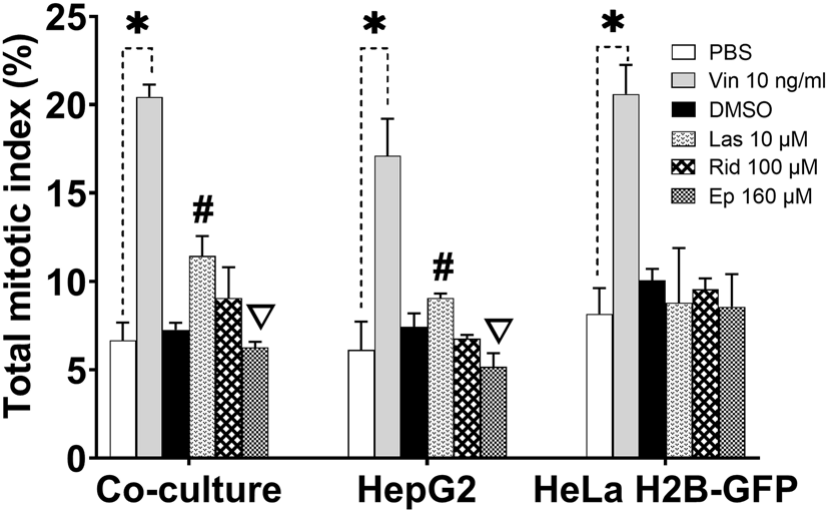
Mitotic index in co-culture, HepG2 and HeLa H2B-GFP cells. Results are presented as mean ± SD of three replicates within the same experiment and *p < 0.05 compared to PBS as solvent control; #*p* < 0.05 compared to DMSO as solvent control; ∇*p* < 0.05 significant decrease effect against DMSO as solvent control. *PBS* solvent control used for vincristine, *DMSO* solvent control used for PAs, *Vin 10 ng/ml* vincristine 10 ng/ml, *Las 10 µM *lasiocarpine 10 µM, *Rid 100 µM*  riddelliine 100 µM, *Ep 160 µM*  europine 160 µM

The microscopic examination of mitotic figures after PA treatment of the co-culture, HepG2 and HeLa H2B-GFP cells was expressed as mitotic index as shown in Fig. 8. Lasiocarpine (10 µM) significantly increased the mitotic index when compared to the solvent control in co-culture and HepG2 only, but did not alter the mitotic index in HeLa H2B-GFP cells if cultured alone. Riddelliine (100 µM) did not cause significant alterations in the mitotic index. Europine (160 µM) significantly decreased the mitotic index in co-culture and HepG2 cells, but caused no change in HeLa H2B-GFP. In summary, there was no difference in the total mitotic index in HeLa H2B-GFP cultured alone and treated with PAs. Our results indicate that PAs require metabolic activation to affect the mitotic processes, as disturbances were observed in HeLa H2B-GFP cells of the co-culture and in HepG2 cells cultured alone, but not in HeLa H2B-GFP cells cultured alone.

**Fig. 9:**
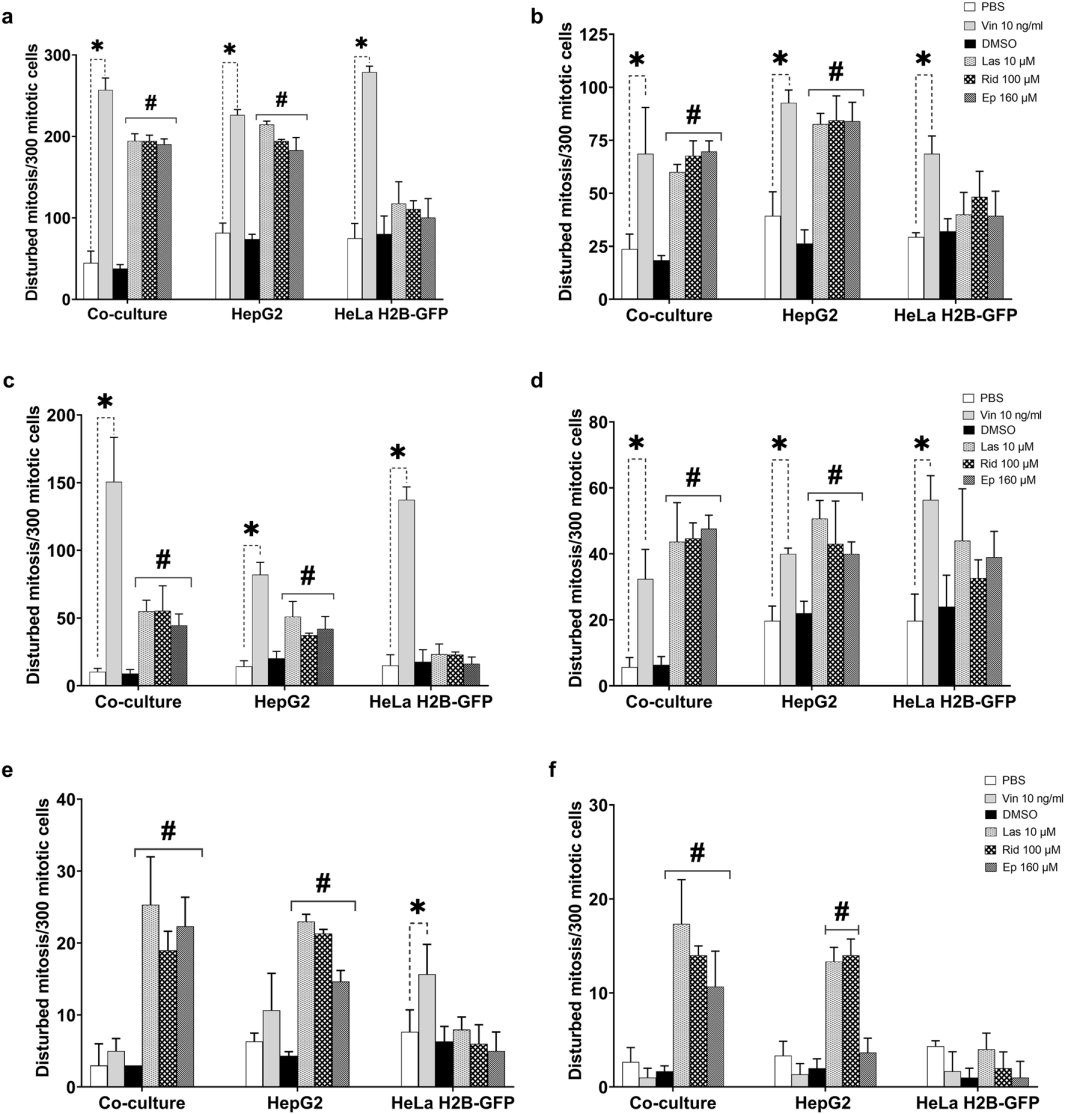
Categories of mitotic disturbances induced by PAs in 300 mitotic cells; HeLa H2B-GFP analysed after co-culture or cultured alone, and HepG2 cells cultured alone. **a** Total mitotic disturbance, **b** Non-congression at metaphase, **c** combination of no-metaphase plate formation and elongated chromosomes at metaphase, **d** multipolar metaphase, anaphase and telophase, **e** lagging chromosome(s)/chromatid(s) at anaphase and telophase and **f** bridges at anaphase and telophase. Results are presented as mean ± SD in three (3) replicates of the same experiment and *p < 0.05 against PBS solvent control; #*p* < 0.05 against DMSO solvent control. *PBS* solvent control used for vincristine, *DMSO* solvent control used for Pas, *Vin 10 ng/ml* vincristine 10 ng/ml, *Las 10 µM* lasiocarpine 10 µM, *Rid 100 µM* riddelliine 100 µM, *Ep 160 µM*  europine 160 µM

The most common chromosomal abnormalities associated with PAs were non-congression, no metaphase-plate formation, bridges, lagging chromosomes and multipolar metaphases, as shown in Fig. 9. The only clear difference between the three used PAs was that the monoester europine induced less ana-/telophase bridges than the two diesters.

## Discussion

The human hepatoma cell line HepG2 cells has been shown to serve as valuable replacements of primary liver cells to study metabolism- and transport-related cellular effects (Gerets et al. 2012). For example, the in vitro study by Louisa et al. (2016) showed that HepG2 cells are sensitive to drug inhibitions of membrane transporters. This enabled us to investigate the effects of inhibitors of metabolism and transporters on the genotoxicity of PAs. In vivo, sinusoidal liver epithelial cells (HSECs) are a very important target for PA-induced toxicity and carcinogenicity, but PAs need to be activated before reaching these cells, because the HSECs are thought to lack cytochrome P450 enzymes for metabolic activation of PA (Chojkier 2003; Edgar et al. 2015; Field et al. 2015; Fu et al. 2004; Gao et al. 2015; Lin et al. 2011; Ruan et al. 2015; Yang et al. 2017). To see whether the PA metabolites formed in hepatocytes may be able to move to the HSEC target cells, we applied a co-culture of HeLa H2B-GFP cells representing metabolically inactive cells and HepG2 cells representing a hepatocyte-like cell type mimicking the events in vitro. Three PAs of different ester types, the open diester lasiocarpine, the cyclic diester riddelliine and the monoester europine induced micronucleus formation significantly in HeLa H2B-GFP in the co-culture, but not if cultured alone. Thus, it can be postulated that the PAs were metabolized by cytochrome P450s in HepG2 cells in the co-culture and then the PA metabolites moved out of the HepG2 cells to react and induce micronucleus formation in non-metabolically active HeLa H2B-GFP cells. The cytochrome P450-3A4 inhibitor ketoconazole reduced the micronucleus induction in HeLa H2B-GFP cells in the co-culture, probably due to inhibition of metabolism in the HepG2 cells. This is in agreement with a recent publication by Ebmeyer et al. (2019), who found that lasciocarpine was only genotoxic in metabolically incompetent V79 hamster cells if they had been genetically engineered to harbour human CYP450-3A4. In agreement with our findings, but not investigating genotoxicity, Lu et al. (2019) found in a two-layer Transwell co-culture model that PAs were metabolized by HepaRG human hepatocyte cells and the generated metabolites reacted with HSECs; the three investigated PAs, retrorsine, monocrotaline and clivorine, induced concentration-dependent cytotoxicity in HSEC (Lu et al. 2019).

In addition to passive diffusion, membrane transporters have a significant role in facilitating or preventing xenobiotic movements (Ho and Kim 2005). They are classified as influx and efflux transporters, which are typically located either at the basolateral or apical membrane in polarized cells such as liver cells (Giacomini and Sugiyama 2015). Therefore, we next used membrane transporter inhibitors, and in the context of the co-culture model the logical choice was to use inhibitors for efflux transporters of presumable metabolites from HepG2 cells. To inhibit influx transporters would disable PAs from entering the HepG2 cells which would be indistinguishable from inhibition of influx of metabolites into HeLa H2B-GFP cells or an inhibition of metabolic enzymes in HepG2 cells. Regarding efflux transporters, it was reported that HepG2 cells express MDR1 (Louisa et al. 2016) and MRP2 (Tocchetti et al. 2018) transporters. Verapamil is a calcium channel blocker and is a specific first-generation MDR1 efflux transporter inhibitor (Donmez et al. 2011; Nobili 2006). Benzbromarone, which is an anti-gout agent, has been shown to inhibit efflux transporter MRP2 (Huang et al. 2018; Jemnitz et al. 2010; Kidron et al. 2012; M. T. Huisman 2010), also in HepG2 cells (Sinclair and Fox 1975). Both efflux membrane transporter inhibitors decreased lasiocarpine-, ridelliine- and europine-induced micronucleus formation in HeLa H2B-GFP cell in the co-culture. This confirms that membrane transporters mediate cellular PA uptake and elimination at least partially, possibly in addition to passive diffusion.

During micronucleus experiments, the disturbance of mitotic figures became apparent and was then investigated in separate experiments. All three PAs caused a variety of mitotic disturbances like non-congression of chromosomes to the metaphase plate, missing metaphase alignment typical for disturbed spindle formation, lagging chromosomes left at the metaphase plate location after separation of the daughter chromosomes, and multipolar metaphases. A well-known mechanism for mitotic disturbance is the inhibition of spindle formation or disassembly. It would be conceivable that PAs react with the tubulin molecule, which harbours many accessible cysteine residues (Mohan et al. 2018; Weber 2015). However, known spindle disturbing substances usually lead to an arrest in metaphase of mitosis (detectable as elevated mitotic index), such as that seen with the positive control and spindle formation inhibitor vincristine, which was only observed to a small extent for lasiocarpine, and not for riddelliine or europine. Therefore, other mechanisms for mitotic disturbance may have to be identified. In a transcriptomics approach, it was recently shown that five PAs (lasiocarpine, riddelliine, lycopsamine, echimidine, and monocrotaline) interfered with cell cycle regulation and DNA damage repair. Furthermore, using microscopic methods, the authors reported chromosome congression defects indicating disturbance of mitosis, which is in agreement with our findings (Abdelfatah et al. 2022).

The only clear difference between the PAs used here was that the monoester europine induced less ana-/telophase bridge formation than the diesters at concentrations which caused a similar number of micronuclei. Different mechanisms for the formation of chromatin bridges have been suggested. One of them is that chromatin bridges between sister chromatids may reveal the presence of cross-links between DNA strands. Due to the presence of a strong mechanical traction during anaphase, covalently bound chromatids can give rise to such anaphase chromatin bridges (Botta and Gustavino 1997; Rizzoni et al. 1993). In our previous study, we found that diester PAs induced DNA cross-links, but the monoester europine did not (Hadi et al. 2021). Thus, the mechanism of mitotic disturbance may be different for monoesters and diesters. A study investigating the fate of anaphase bridges in cultured oral squamous cell carcinoma cells in real time revealed that chromosomes in bridges typically resolve by breaking into multiple fragments. Often, these fragments give rise to micronuclei (MN) at the end of mitosis (Hoffelder et al. 2004). However, since the induced micronucleus frequency was similar for the three PAs under the applied conditions, bridge formation may only contribute a small number of micronuclei to the overall frequency. Support for a difference between monoester and open or cyclic diester PAs comes from a study in which effective metabolic degradation by human liver microsomes was observed for diesters, but not for monoesters (Geburek et al. 2020) and a paper in which the cyclic diester riddelliine was described as a more potent DNA cross-linker than heliosupine, which is an open diester (Hincks et al. 1991).

Concentrations applied here are orders of magnitude higher than the average human exposure. However, metabolic activation may be more efficient in vivo and accumulation of mutagenic adducts in the liver may occur. The elucidation of mechanisms of action is therefore relevant for human risk assessment.

In conclusion, the co-culture of HepG2 cells with HeLa H2B-GFP cells helped to further support the role of metabolic activation, to show that metabolites can reach another, metabolically inactive cell type in the vicinity of the metabolically active cells, and that efflux transporters enable or support the movement of metabolites. Regarding the mode of action, in addition to the reactive intermediates forming DNA adducts, which then lead to mutations, we describe that mitotic disturbances are induced to a large extent. Mitotic disturbances may lead to or contribute to the micronucleus formation caused by PAs.

## Data Availability

Available upon request.
